# Correlation between Serum Bone Turnover Markers and Estimated Glomerular Filtration Rate in Chinese Patients with Diabetes

**DOI:** 10.1155/2021/6731218

**Published:** 2021-01-06

**Authors:** Xuming Zhu, Ying Zhou, Shanchao Hong, Yifeng Xue, Yubao Cui

**Affiliations:** Department of Clinical Laboratory, Wuxi People's Hospital Affiliated with Nanjing Medical University, Wuxi, Jiangsu Province, China

## Abstract

**Objective:**

Diabetes is a growing global public health concern with many significant disease complications. Multiple studies show that bone turnover markers (BTMs) are decreased in diabetes patients, indicating impaired bone metabolism in diabetes patients. A recent study also showed that in diabetes patients, BTMs are correlated with urine albumin to creatinine ratio, an indicator of nephropathy. However, whether BTMs are correlated with estimated glomerular filtration rate (eGFR) in diabetes remains unknown. This retrospective study accessed correlations between serum BTMs and eGFR in Chinese patients with diabetes and compare levels of BTMs and eGFR between diabetic patients and healthy individuals.

**Methods:**

This study analyzed data from 221 diabetic patients (include type1 and type 2 diabetes) and 155 healthy individuals. Serum BTM levels and eGFR were compared between diabetic patients and healthy individuals. Pearson correlation analysis was used to assess correlations between BTMs and eGFR. Multiple logistic regression analysis adjusted for gender and age was performed to measure odd ratio (OR) and 95% confidence interval (95% CI) of BTMs on diabetes.

**Results:**

Patients with diabetes had significant lower 25-hydroxyvitamin D (25(OH)D) levels (15.07 ± 6.20 ng/mL) than healthy group (17.89 ± 6.41 ng/mL) (*P* < 0.05). For patients with diabetes, eGFR was negatively correlated with osteocalcin (OC) (*r* = −0.434, *P* < 0.05), procollagen type 1 intact N-terminal propeptide (P1NP) (*r* = −0.350, *P* < 0.05), and *β*-carboxy-terminal cross-linking telopeptide of type I collagen (*β*-CTX) (*r* = −0.179, *P* < 0.05) levels. For healthy people, eGFR was negatively correlated with 25(OH)D (*r* = −0.290, *P* < 0.05) levels. Multiple logistic regression analysis adjusted for age and gender (mean age of diabetes was 64.9 years and the percentage of female was 66.9%, mean age of healthy people was 48.4 years and the percentage of female was 37.4%) showed that 25(OH)D (OR = 0.909, 95%CI = 0.862 − 0.959, *P* < 0.05) was protective factors for diabetes.

**Conclusions:**

In the stage of diabetic nephropathy, bone turnover may accelerate. It is important to detect BTMs in the stage of diabetic nephropathy.

## 1. Introduction

There were an estimated 415 million people aged 20-79 years with diabetes worldwide, contributing to an estimated expenditure of $673 billion due to diabetes in 2015 [1]. Further, the number of people aged 20-79 years with diabetes is predicted to rise to 642 million worldwide by 2040 [1]. In China, about one in five people aged over 50 years has diabetes [2]. Therefore, diabetes is a significant public health concern and clinical burden. The most common complications of diabetes include macrovascular disease, retinopathy, nephropathy, and peripheral neuropathy [3]. Recent research shows that bone strength is also impaired in type 2 diabetic patients, and patients with diabetes have an increased risk for fractures [4]. Furthermore, bone impairment starts in childhood with type 1 diabetes [5, 6]. Type 2 diabetes disrupts bone cell function, variably impacting bone formation, turnover, and resorption [4]. Further, diabetes mellitus and bone disease have numerous epidemiological and pathophysiological associations in common [7].

Bone turnover markers (BTMs) are markers to reflect bone metabolism, such as 25-hydroxyvitamin D (25(OH)D), osteocalcin (OC), procollagen type 1 intact N-terminal propeptide (P1NP), and *β*-carboxy-terminal cross-linking telopeptide of type I collagen (*β*-CTX). BTMs indicate dynamics of bone turnover and correlate with osteoporosis and fracture [8, 9]. A meta-analysis has shown markers of both bone formation and bone resorption are decreased in patients with diabetes, suggesting diabetes mellitus is a state of impaired bone metabolism [10].

Diabetic nephropathy is one of the most important diabetic microvascular complications, affecting 30%-45% patients with diabetes, and is the leading cause of chronic kidney disease and end-stage renal disease worldwide [11, 12]. Considering progressive renal functional impairment resulting from diabetic nephropathy, timely detection of renal impairment in diabetes is important. Estimated glomerular filtration rate (eGFR) is commonly used to define, classify, screen, and monitor chronic kidney disease [13]. Although there are novel markers to detect chronic kidney disease in diabetes at present [14, 15], eGFR is still widely used to assess the renal status in diabetes [16].

A recent study has shown that in diabetes, BTMs correlate with urine albumin to creatinine ratio, an indicator of early-stage nephropathy [17]. However, whether in diabetes BTMs are correlated with eGFR, a more commonly used marker to monitor chronic kidney disease, remains unknown. Therefore, this retrospective study accesses correlations between serum BTMs and eGFR in Chinese patients with diabetes as well as differences of BTMs and eGFR between diabetic patients and healthy people.

## 2. Materials and Methods

### 2.1. Study Population

This retrospective study analyzed data from 376 participants treated in Wuxi People's Hospital Affiliated with Nanjing Medical University between January 2019 and July 2019. The diabetes group included 221 both type 1 and type 2 diabetic patients treated in the endocrinology department, and the control group included 155 healthy individuals seen in physical examination center. Diabetes was diagnosed according to 2010 World Health Organization criteria. The exclusion criteria for participants included malignancy, chronic obstructive pulmonary disease, depression diseases, and chronic or recent use of drugs with an action on bone metabolism. This study was approved by the ethics committee of Wuxi People's Hospital Affiliated with Nanjing Medical University. Patient consent was waived due to the retrospective nature of this study. The data was maintained with confidentiality, and this study was conducted in accordance with the Declaration of Helsinki.

### 2.2. Laboratory Assays

After fasting for at least 8 hours, 10 mL venous blood from each participant was drawn into vacuum tubes. Blood samples were centrifuged at 3000 x g for 3 min, and serum or plasma was collected. Within 2 hours, a Beckman AU5800 Automatic Analyzer (Beckman Coulter Inc., CA, USA) was used to measure serum index: electrolyte, including potassium (K), sodium (Na), chlorine (Cl), calcium (Ca), and carbon dioxide (CO_2_); liver function, including total bilirubin (Tbil), total protein (TP), albumin (ALB), alkaline phosphatase (ALP), and alanine transaminase (ALT); and renal function, including urea nitrogen (BUN), creatinine (Cr), and glucose (Glu). BTMs from plasma, including 25(OH)D, OC, P1NP, and *β*-CTX, were measured by electrochemical luminescence immunoassay on an automatic Roche Cobas E601 analyser (Roche Diagnostics GmbH, Mannheim, Germany). Plasma was stored at 4°C no more than 48 hours before measurement.

### 2.3. Calculations of eGFR

There are several equations to calculate eGFR [18]. According to the CKD-EPI-ASIA equation [17], we calculated eGFR using serum Cr levels.

For males:

Cr ≤ 0.9 mg/dL: eGFR = 141 × (sCr/0.9)^−0.411^ × 0.993^age^ × 1.057

Cr > 0.9 mg/dL: eGFR = 141 × (sCr/0.9)^−1.209^ × 0.993^age^ × 1.057

For females:

Cr ≤ 0.7 mg/dL: eGFR = 141 × (sCr/0.7)^−0.329^ × 0.993^age^ × 1.049

Cr > 0.7 mg/dL: eGFR = 141 × (sCr/0.7)^−1.209^ × 0.993^age^ × 1.049

Patients with diabetes were divided into two groups according to eGFR levels: low eGFR group (<90 mL/min) and high eGFR group (≧90 mL/min).

### 2.4. Statistical Analysis

All statistical analyses were performed using SPSS version 20.0 (SPSS Inc., Chicago, USA). Continuous data were presented as mean ± standard deviation. The Kolmosprov-Smirnov test was used to measure the normal distribution of data. If data had a normal distribution, Student's *t*-test was used to compare differences of continuous data in different groups. Otherwise, Mann Whitney *U*-test was performed. Male/female ratio was compared by chi-squared test. Pearson correlation analysis was used to assess correlations between BTMs and eGFR. Multiple logistic regression analysis adjusted for gender and age was performed to measure odd ratio (OR) and 95% confidence interval (95% CI) of BTMs on diabetes. Values of *P* < 0.05 were considered statistically significant.

## 3. Results

### 3.1. General Characteristics of Diabetes and Normal Groups

All participants aged from 22 to 93 years. As shown in Table 1, diabetes and normal groups had significantly different male/female ratio, age, K, Cl, Ca, CO_2,_ TBIL, TP, ALB, ALT, Glu, and eGFR (*P* < 0.05). The diabetes group had significantly decreased eGFR compared to normal group (*P* < 0.05). No significant differences between groups were detected in Na, ALP, BUN, and Cr (*P* > 0.05).

### 3.2. BTMs of Diabetes and Normal Groups

BTM levels in diabetes and normal groups were shown in Table 2. The only significant difference was lower 25(OH)D levels in the diabetes group compared to the normal group (*P* < 0.05).

For diabetes patients (Table 3), 25(OH)D levels were significantly decreased in the low eGFR group compared to the high eGFR group (*P* < 0.05), while OC, P1NP, and *β*-CTX levels were significantly increased in the low eGFR group compared to the high eGFR group (*P* < 0.05).

### 3.3. Univariate Correlation Analysis of BTMs with eGFR

As shown in Table 4, eGFR had significantly negative correlation with OC (*r* = −0.434, *P* < 0.05), P1NP (*r* = −0.350, *P* < 0.05), and *β*-CTX levels (*r* = −0.179, *P* < 0.05) but not 25(OH)D levels (*P* > 0.05) in patients with diabetes. Further, eGFR had significantly negative correlation with only 25(OH)D levels (*r* = −0.290, *P* < 0.05) in normal group.

### 3.4. Multiple Logistic Regression Analysis

There were significant differences of male/female ratio and age between patients with diabetes and healthy individuals, so a multiple logistic regression analysis should be performed with adjusted for gender and age. Multiple logistic regression analysis adjusted for gender and age (Table 5) showed that 25(OH)D (OR = 0.909, 95%CI = 0.862 − 0.959, *P* < 0.05) was significantly associated with diabetes while eGFR, OC, P1NP, and *β*-CTX were not (*P* > 0.05).

## 4. Discussion

This study showed that among serum BTMs, only 25(OH)D decreased in diabetes group while OC, P1NP, and *β*-CTX levels had no significant differences between diabetes and normal groups. Conversely, a previous study showed that men over the age of 50 years with type 2 diabetes had higher 25(OH)D levels and lower OC, P1NP, and *β*-CTX levels than normal group [19]. Further, a meta-analysis in 2014 showed OC and *β*-CTX decreased in diabetic patients compared to nondiabetic controls [20]. In present study difference of OC, P1NP, and *β*-CTX could not be shown due to the mixed population of diabetes patients (type 1 and type 2) and the gender/age differences in groups, which were really confound variables.

In diabetes group, our results demonstrated that 25(OH)D levels decreased in the low eGFR group (<90 mL/min) compared to the high eGFRs group (≧90 mL/min). Previous study showed vitamin D deficiency was positively associated with decreased eGFR in Korean adults [21]. High level of vitamin D may be related with chronic kidney disease. On the contrary, OC, P1NP, and *β*-CTX levels increased in low eGFR group. Further, OC, TP1NP, and *β*-CTX levels negatively correlated with eGFR in diabetes patients while 25(OH)D negatively correlated with eGFR in normal people. To our knowledge, this is the first study concerning correlations between BTMs and eGFR in diabetes. The increase of urine albumin to creatinine ratio is the main clinical manifestation to reflect early-stage kidney changes, and a recent study has revealed that urine albumin to creatinine ratio is positively correlated with OC, P1NP, and *β*-CTX while negatively correlated with 25(OH)D in type 2 diabetes patients [17]. These conclusions have confirmed BTMs are positively correlated with markers reflecting severity of renal impairment in diabetes patients, suggesting that in the stage of diabetic nephropathy, bone turnover may be accelerated. It is important to detect BTMs in the stage of diabetic nephropathy.

As a form of vitamin D, 25(OH)D was proved to be associated with diabetes based on our logistic regression analysis. Similarly, a recent meta-analysis has shown an inverse and significant association between broad range 25(OH)D levels and risk of type 2 diabetes in diverse populations [22]. A mechanism for 25(OH)D to exert effect on diabetes is that insulin secretion may be influenced by vitamin D indirectly with a vitamin D-dependent Ca-binding protein in pancreatic *β* cells [23]. Our logistic analysis also showed that other BTMs including OC, P1NP, and *β*-CTX had no effect on diabetes due to limitations of retrospective nature of this paper, mixed populations of type 1 and type 2 diabetes, and differences in gender/age between groups. However, a meta-analysis in 2015 implied that OC could influence blood glucose [24], contrary to our conclusion. A previous study indicated that genetic factors might play a role in modulating OC and glucose metabolism in different ethnic population [25]. Therefore, more comprehensive meta-analyses with more related publications will be needed in the future.

This retrospective study also has some limitations. First, we did not divide diabetes into type 1 and type 2 disease, ignoring their different pathogenic mechanisms. Second, we did not evaluate therapy measures concerning glycemic control, osteoporosis, and diabetic nephropathy, which could influence BTM and eGFR levels to some extent.

In conclusion, this study has found that OC, P1NP, and *β*-CTX levels are negatively correlated with eGFR in diabetes patients while 25(OH)D is negatively correlated with eGFR in healthy people, suggesting that in the stage of diabetic nephropathy, bone turnover may accelerate. Further studies are needed to evaluate whether preventing the progression of diabetic nephropathy can influence bone turnover.

## Figures and Tables

**Table 1:** General characteristics of diabetes and normal groups.

Index	Diabetes group (*n* = 221)	Control group (*n* = 155)	*P* value
Male/female (*n*)	73/148	97/58	<0.01
Age (years)	64.90 ± 11.23	48.44 ± 10.43	<0.01
K (mmol/L)	3.82 ± 0.37	4.05 ± 0.28	<0.01
Na (mmol/L)	140.23 ± 2.52	140.52 ± 1.91	0.232
Cl (mmol/L)	105.11 ± 2.83	104.06 ± 2.00	<0.01
Ca (mmol/L)	2.31 ± 0.12	2.34 ± 0.09	<0.01
CO_2_ (mmol/L)	26.61 ± 2.43	25.12 ± 1.83	<0.01
TBIL (*μ*mol/L)	12.36 ± 4.97	14.00 ± 4.77	<0.01
TP (g/L)	67.90 ± 7.67	73.49 ± 3.89	<0.01
ALB (g/L)	38.70 ± 4.35	43.83 ± 2.51	<0.01
ALP (U/L)	83.84 ± 34.19	81.17 ± 24.64	0.380
ALT (U/L)	20.38 ± 12.66	24.49 ± 15.19	<0.01
BUN (mmol/L)	5.66 ± 2.23	5.35 ± 1.27	0.084
Cr (*μ*mol/L)	68.36 ± 48.77	67.06 ± 13.86	0.707
Glu (mmol/L)	7.86 ± 3.08	5.68 ± 1.76	<0.01
eGFR (mL/min)	95.37 ± 20.61	108.44 ± 11.94	<0.01

TBIL: total bilirubin; TP: total protein; ALB: albumin; ALP: alkaline phosphatase; ALT: alanine transaminase; BUN: urea nitrogen; Cr: creatinine; Glu: glucose; eGFRs: estimated glomerular filtrations rates.

**Table 2:** Bone turnover markers of diabetes and normal groups.

Marker	Diabetes group (*n* = 221)	Control group (*n* = 155)	*P* value
25(OH)D (ng/mL)	15.07 ± 6.20	17.89 ± 6.41	<0.01
OC (ng/mL)	15.38 ± 11.13	16.57 ± 5.49	0.222
P1NP (ng/mL)	43.28 ± 23.95	46.12 ± 18.04	0.213
*β*-CTX (pg/mL)	408.42 ± 219.98	434.08 ± 193.69	0.243

25(OH)D: 25-hydroxyvitamin D; OC: osteocalcin; P1NP: procollagen type 1 intact N-terminal propeptide; *β*-CTX: *β*-carboxy-terminal cross-linking telopeptide of type I collagen.

**Table 3:** Bone turnover markers of low and high eGFR groups in diabetes.

Marker	eGFR < 90 mL/min (*n* = 61)	eGFR≧90 mL/min (*n* = 160)	*P* value
25(OH)D	13.42 ± 5.90	15.69 ± 6.22	0.015
OC	18.98 ± 18.78	14.01 ± 5.61	0.046
P1NP	52.40 ± 36.10	39.79 ± 16.04	0.011
*β*-CTX	459.34 ± 234.31	389.01 ± 211.82	0.033

**Table 4:** Correlation analysis of BTMs with eGFR.

Marker	All participant (*n* = 376)		Diabetes group (*n* = 221)		Control group (*n* = 155)	
	*r*	*P* value	*r*	*P* value	*r*	*P* value
25(OH)D	0.032	0.532	0.054	0.428	-0.290	<0.01
OC	-0.334	<0.01	-0.434	<0.01	-0.116	0.152
P1NP	-0.247	<0.01	-0.350	<0.01	-0.091	0.259
*β*-CTX	-0.129	0.012	-0.179	0.008	-0.115	0.153

**Table 5:** Multivariate logistic regression analysis adjusted for gender and age.

Index	*B*	OR	95% CI	*P* value
25(OH)D	-0.095	0.909	0.862-0.959	<0.01
OC	0.016	1.017	0.954-1.083	0.610
P1NP	-0.023	0.977	0.949-1.006	0.125
*β*-CTX	0.00	1.000	0.998-1.002	0.717
eGFR	-0.004	0.996	0.965-1.028	0.806

## Data Availability

This is a restropective paper. The data used to support the findings of this study are available from the corresponding author upon request.
